# Gastric Lipoma Presenting with Massive Upper Gastrointestinal Bleeding

**DOI:** 10.1155/2013/506101

**Published:** 2013-12-02

**Authors:** Michael J. Ramdass, Sanjana Mathur, Panduranga Seetahal-Maraj, Shaheeba Barrow

**Affiliations:** Department of Surgery, General Hospital, Port-of-Spain, Trinidad, Trinidad And Tobago

## Abstract

A case of massive upper gastrointestinal bleeding in a 37-year-old female is presented showing a submucosal mass in the gastric body. At laparotomy a pedunculated submucosal mass was found located on the posterior wall at the junction of the body and antrum of the stomach, 8 cm from the pylorus. Pathology confirmed that it was a 4 cm benign gastric lipoma with a bleeding central ulcer. Gastric lipomas are rare, benign, typically submucosal tumors occurring in the gastric antrum. They are usually asymptomatic but can become symptomatic depending on size, location, and if there is ulceration of the lesion. These lesions may be mistaken as malignant tumors or present with upper GI bleeding or intussusception. The diagnosis can be made using a combination of upper endoscopy, endoscopic ultrasound, CT, and MRI with surgical excision being the definitive treatment of choice. We hope that this case highlights the fact that these lesions can present with massive upper GI haemorrhage and should be included in the diagnosis when appropriate.

## 1. Introduction

Gastric lipomas are rare and account for less than 1% of all tumors of the stomach and 5% of all gastrointestinal lipomas [1, 2]. They typically occur in the 5th or 6th decade of life with equal sex incidences and 75% occur in the antral region in the submucosa or serosal layers [3]. They are usually asymptomatic and are commonly detected incidentally; however, they may present with gastric outlet obstruction and upper gastrointestinal bleeding. Approximately 220 cases have been reported in the medical literature and further only two cases have been reported presenting with massive upper gastrointestinal haemorrhage [4, 5].

## 2. Case Report

A 37-year-old female presented with a four-day history of epigastric pain and melaena associated with vomiting. The pain was sudden in onset, severe, and with no radiation. There were no aggravating factors and it was associated with three episodes of black, tarry, and foul-smelling stool. She had a history of weakness, dyspnoea, headaches, and palpitations (symptoms of anaemia) and used Ibuprofen for menstrual cramps on a monthly basis. There was no history of cigarette smoking, alcohol use, peptic ulcer disease, or reflux. Examination revealed pale mucous membranes and epigastric tenderness. The haemoglobin dropped to 5.9 g/dL and she was transfused 6 units of packed cells.

**Figure 1:**
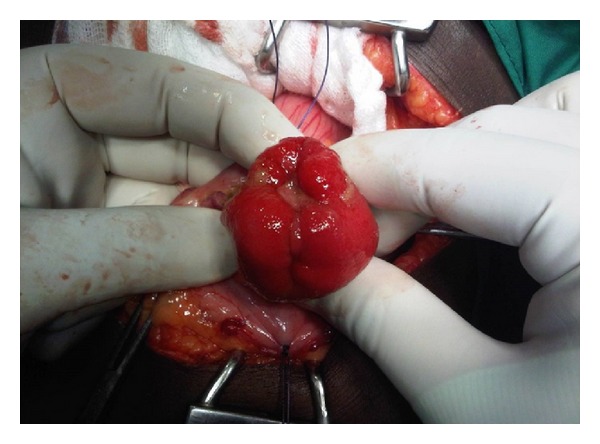
Intraoperative photo of gastric lipoma with central ulcer bleeding.

**Figure 2:**
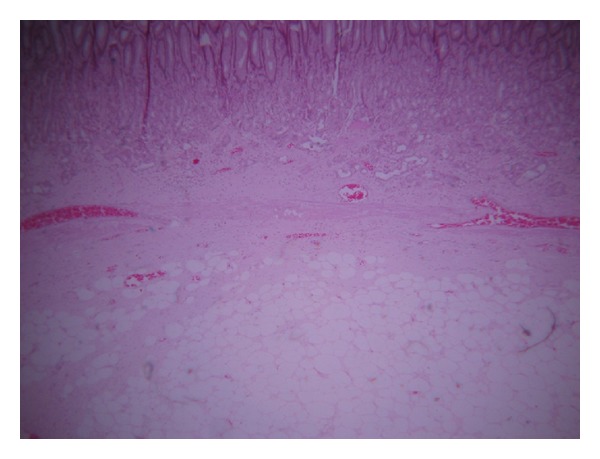
Photomicrograph showing gastric mucosa with submucosal adipocytes, confirming lipoma.

An upper GI endoscopy revealed a normal oesophagus, cardia, and fundus with a submucosal mass with a 1 cm ulcerating area in the gastric body. The patient was prepared for a laparotomy and an anterior gastrotomy was performed on the basis of the current diagnosis of a stromal tumor. A pedunculated submucosal mass was found, located on the posterior wall at the junction of the body and antrum of the stomach, 8 cm from pylorus (Figure 1). Pathologic findings included a gross specimen measuring 4 × 3.5 × 3.2 cm on cross-section. It was spherical and yellow in colour and histology confirmed the lesion to be a benign gastric lipoma with a central ulcer (Figure 2). The patient had an uneventful recovery and did well postoperatively.

## 3. Discussion

Gastrointestinal (GI) lipomas are benign tumors composed of mature adipose tissue covered with a fibrous capsule. Most GI lipomas are located in the colon, ileum, and jejunum and are predominantly asymptomatic. Gastric lipomas consist of less than 1 percent of all benign gastric tumors and 5 percent of all GI lipomas [1, 2]. They are typically found in patients in the 50–60 age range, but cases have been reported in significantly younger persons, with a total of 6 reported paediatric cases [6].

Most lipomas are found in the submucosa (95%), the subserosal subtype being extremely rare. They are usually solitary and most commonly found in the antrum, with an incidence of 75% [3]. Although predominantly asymptomatic and indolent, they may be symptomatic owing to size and ulceration. It is reported that a lipoma of size greater than 2.0 cm will present with abdominal pain more than 50% of the time; however, 37% of patients will have a presentation of chronic or acute GI bleeding, obstruction, and dyspepsia [7, 8]. Reports discuss ulceration with necrosis and inflammation as frequent findings; however, gastroduodenal intussusception and massive upper GI bleeding are rarely seen. One report notes that it can present as both a diagnostic problem and as a life threatening lesion due to exsanguinating hemorrhage. The differential diagnosis includes peptic ulcer disease, stromal tumor, liposarcoma, fibroma, or a glomus tumor. The diagnosis is made by endoscopy and radiology [7, 8].

Upper GI endoscopy will show a submucosal mass and three signs which help diagnosis [9]. These are the tenting sign, the cushion sign, and the naked fat sign and are characteristics of gastric lipomas. The tenting sign occurs when the overlying mucosa can be easily retracted with the biopsy forceps, the cushion sign is demonstrated when the forceps makes a soft, cushioning indentation when pressed against the lipoma, and the naked fat sign is visible, exposed adipose tissue on the surface of the lipoma that is projected through the normal overlying mucosa after multiple biopsies of the normal mucosa are done [9]. Endoscopic biopsy is usually inconclusive since the tumour is frequently submucosal.

Computed tomography has been shown to be valuable for diagnosis, demonstrating a well-circumscribed, submucosal mass with uniform fat density and attenuation ranging between −70 and −120 Hz. CT scanning of large (>2 cm) submucosal gastric masses detected on endoscopy can obviate the need for biopsy. Magnetic resonance imaging may show high signal intensity on T1 weighted sequences typical of a lipoma. Endoscopic ultrasound is also quite useful and may show a hyperechoic and isodense mass as opposed to a fibrolipoma which is hyperechoic and heterogenous [10].

The histology in this particular case showed a tumor composed of mature adipocyte proliferation, showing significant variation in cell size, associated with some lipoblasts. Some nuclei were large, slightly irregular but without hyperchromasia or mitosis. Grossly, a lipoma with ulceration, haemorrhage, acute, and chronic inflammation was seen (Figure 2). The diagnosis of a well-differentiated liposarcoma was suspected but molecular cytogenetic analyses showed no MDM2 or CDK4 gene amplification on fluorescent in situ hybridization [9] and the diagnosis of a benign lipoma was confirmed.

Significant complications of a symptomatic gastric lipoma include gastrointestinal obstruction, gastroduodenal intussusception, and severe massive GI haemorrhage. Very rarely, these tumors can become malignant, with a handful of cases being reported in the literature. Histologically, there are four types of liposarcomas including well-differentiated, myxoid, round cell, and pleomorphic. Well-differentiated liposarcomas account for 40% of all liposarcomas, having a peak incidence between the 5th and 7th decades and are further subdivided into adipocytic, sclerosing, inflammatory, and spindle cell subtypes [11].

In conclusion gastric lipomas are benign, typically submucosal tumors occurring in the gastric antrum. They are usually asymptomatic but can become symptomatic depending on size, location, and if there is ulceration of the lesion. These lesions may be mistaken as malignant tumors or present with upper GI bleeding or intussusception. The diagnosis can be made using a combination of upper endoscopy, endoscopic ultrasound, CT, and MRI with surgical excision being the definitive treatment of choice. We hope that this case highlights the fact that these lesions can present with massive upper GI haemorrhage and should be included in the diagnosis when appropriate.
